# Supplementary material for: Genetic tracing of market wildlife and viruses at the epicenter of the COVID-19 pandemic
Source: bioRxiv. 2023 Sep 14:2023.09.13.557637. Preprint. [Version 1] doi: 10.1101/2023.09.13.557637 (PMC10515900; doi:10.1101/2023.09.13.557637)

bioRxiv preprint doi: https://doi.org/10.1101/2023.09.13.557637; this version posted September 14, 2023. The copyright holder for this preprint (which was not certified by peer review) is the author/funder, who has granted bioRxiv a license to display the preprint in perpetuity. It is made available under aCC-BY 4.0 International license.

Supplementary Figures

Figure S1: A. SARS-CoV-2 positivity by stall for samples collected on January 1st and 12th, and B. market sampling map.

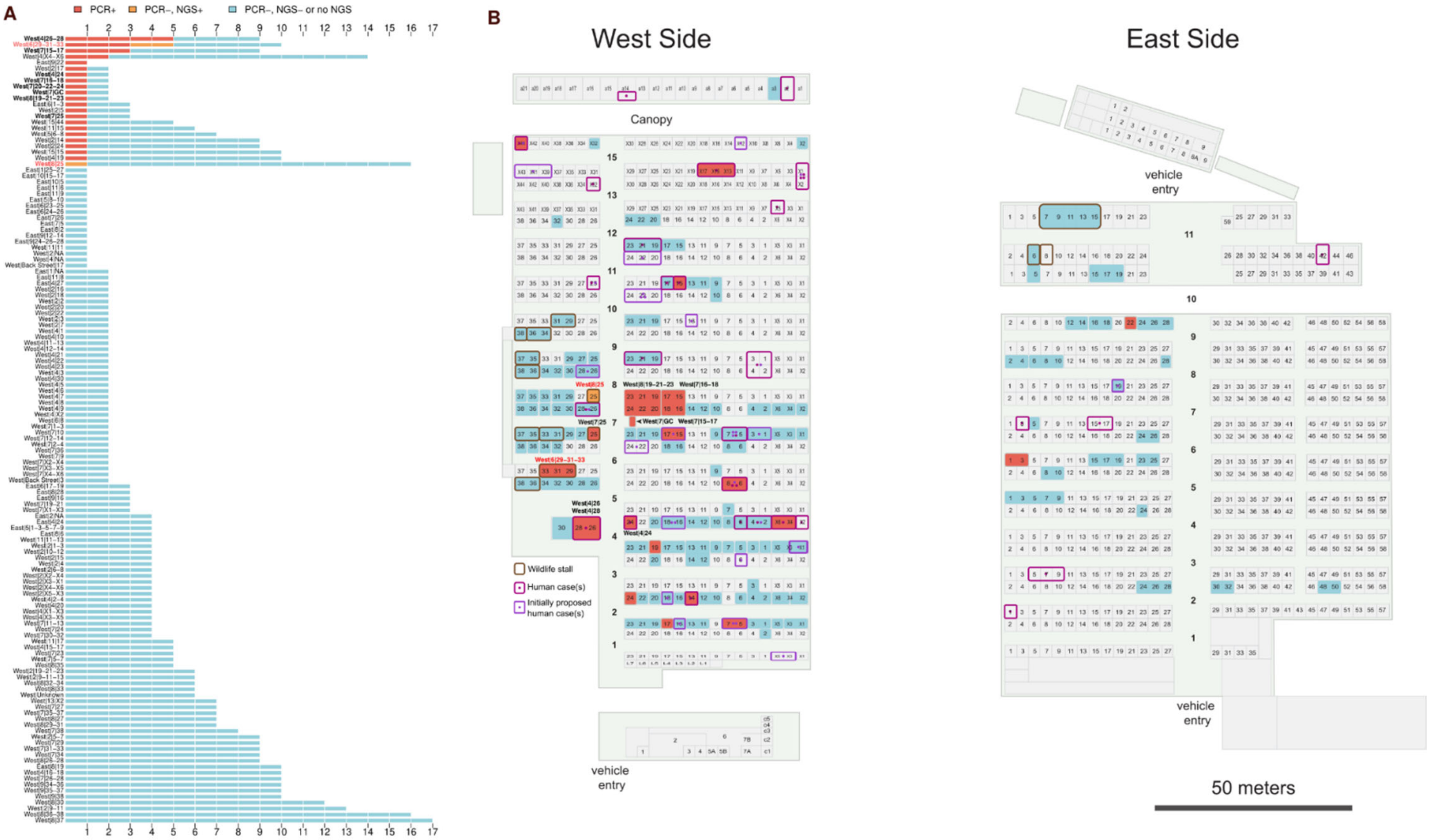

bioRxiv preprint doi: https://doi.org/10.1101/2023.09.13.557637; this version posted September 14, 2023. The copyright holder for this preprint (which was not certified by peer review) is the author/funder, who has granted bioRxiv a license to display the preprint in perpetuity. It is made available under aCC-BY 4.0 International license.

Figure S2: SARS-CoV-2 qPCR Ct values by date of sampling. *: Ct values were not recorded by Liu et al. for the January 12th samples (3 were positive by qPCR). Two Ct values are available for the February 20th sample. Data from Liu et al. Supplementary Table 2.

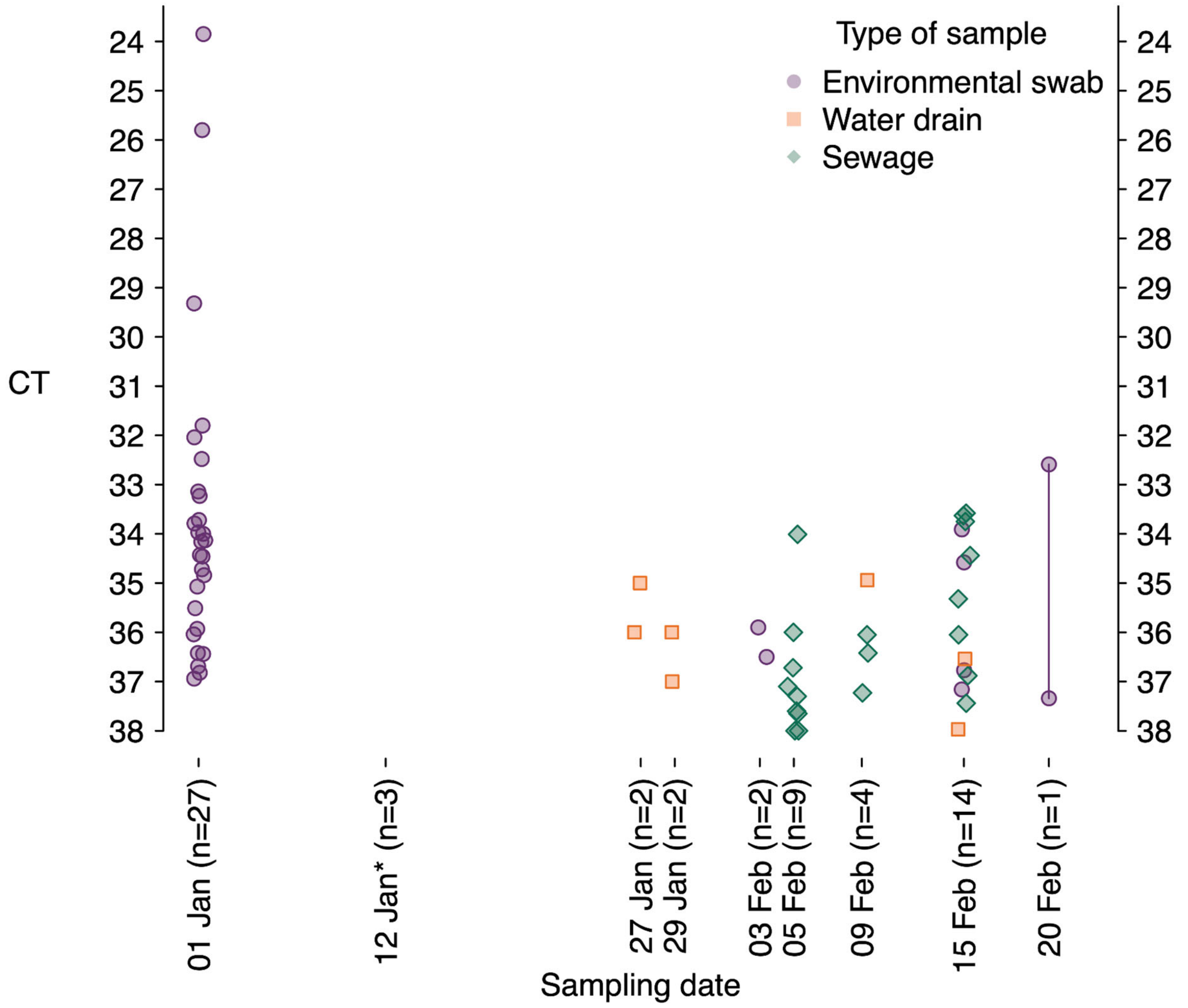

bioRxiv preprint doi: https://doi.org/10.1101/2023.09.13.557637; this version posted September 14, 2023. The copyright holder for this preprint (which was not certified by peer review) is the author/funder, who has granted bioRxiv a license to display the preprint in perpetuity. It is made available under aCC-BY 4.0 International license.

Figure S3: Abundances of mitochondrial DNA from humans and key wildlife species in SARS-CoV-2 positive (top row) and negative (bottom row) samples from Stall 6-29 with and without including mitochondrial rRNA regions.

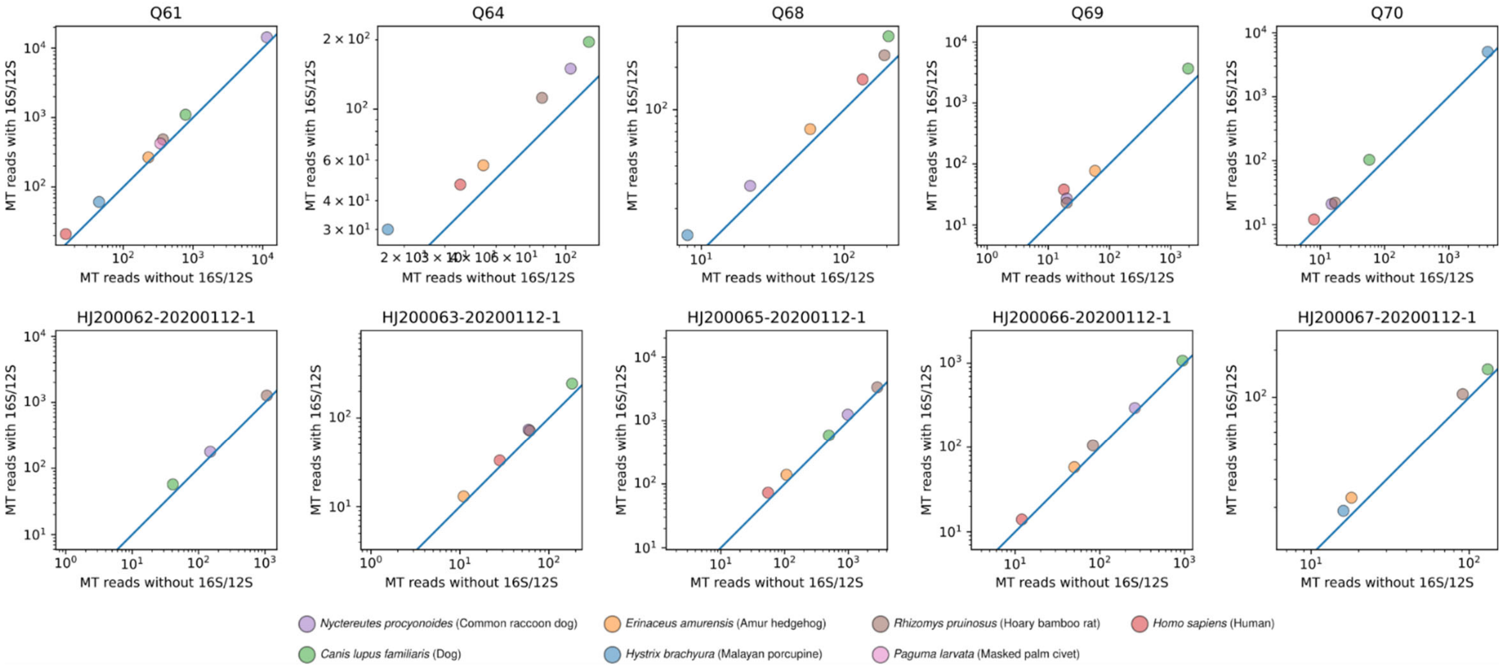

bioRxiv preprint doi: https://doi.org/10.1101/2023.09.13.557637; this version posted September 14, 2023. The copyright holder for this preprint (which was not certified by peer review) is the author/funder, who has granted bioRxiv a license to display the preprint in perpetuity. It is made available under aCC-BY 4.0 International license.

Figure S4: Spearman correlations between animal species abundances and SARS-CoV-2 reads in different sample sets. The estimated Spearman correlation coefficient and its 95% CI are shown for species detected in 3 or more samples collected on January 1st, January 12th, or either date. Highlighted points have uncorrected p-values below 0.05.

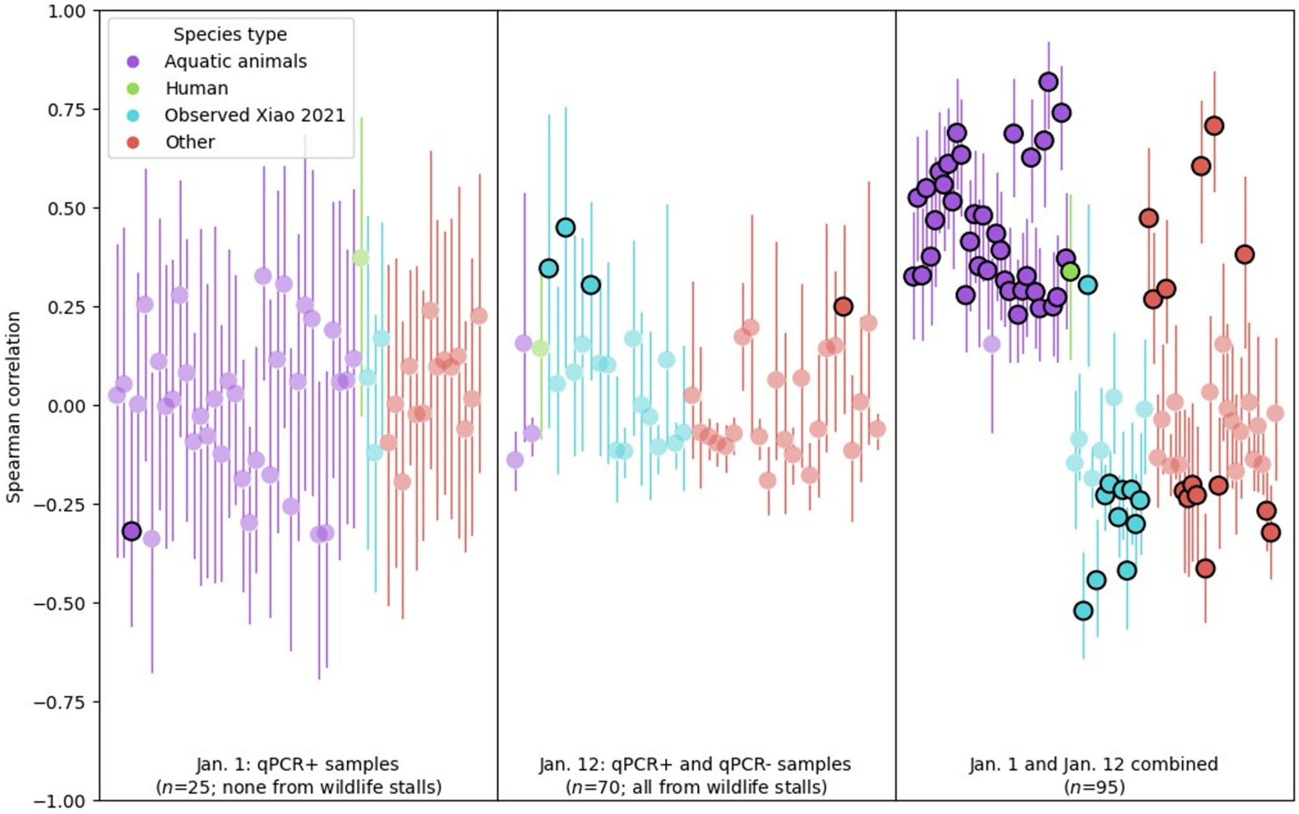

bioRxiv preprint doi: https://doi.org/10.1101/2023.09.13.557637; this version posted September 14, 2023. The copyright holder for this preprint (which was not certified by peer review) is the author/funder, who has granted bioRxiv a license to display the preprint in perpetuity. It is made available under aCC-BY 4.0 International license.

Figure S5: Viral abundances within 5 SARS-CoV-2 positive samples from a wildlife stall.

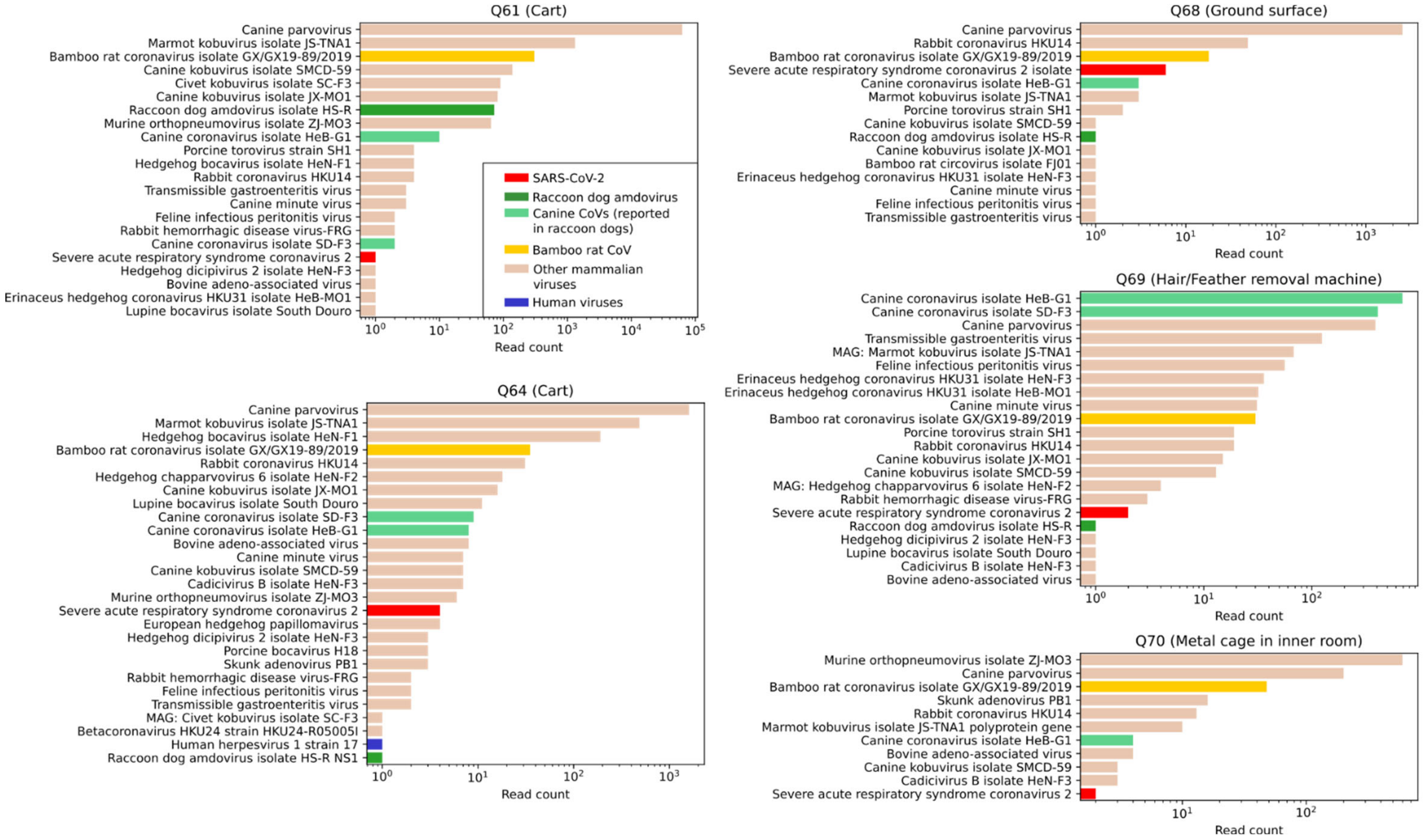

Supplement: 2 [file NIHPP2023.09.13.557637v1-supplement-2.pdf]
